# Altered trigeminothalamic spontaneous low-frequency oscillations in migraine without aura: a resting-state fMRI study

**DOI:** 10.1186/s12883-021-02374-7

**Published:** 2021-09-07

**Authors:** Ye Eun Kim, Min Kyung Kim, Sang-il Suh, Ji Hyun Kim

**Affiliations:** 1 Department of Neurology, Korea University Guro Hospital, Korea University College of Medicine, 152-703, Guro-dong gil 97, Guro-dong, Guro-gu, Seoul, Republic of Korea; 2 Department of Radiology, Korea University Guro Hospital, Korea University College of Medicine, Seoul, South Korea

**Keywords:** Migraine without aura, Low-frequency oscillation, Fractional amplitude of low-frequency fluctuation, Brainstem, Thalamus

## Abstract

**Background:**

Recent resting-state fMRI studies demonstrated functional dysconnectivity within the central pain matrix in migraineurs. This study aimed to investigate the spatial distribution and amplitude of low-frequency oscillations (LFOs) using fractional amplitude of low-frequency fluctuation (fALFF) analysis in migraine patients without aura, and to examine relationships between regional LFOs and clinical variables.

**Methods:**

Resting-state fMRI data were obtained and preprocessed in 44 migraine patients without aura and 31 matched controls. fALFF was computed according to the original method, *z*-transformed for standardization, and compared between migraineurs and controls. Correlation analysis between regional fALFF and clinical variables was performed in migraineurs as well.

**Results:**

Compared with controls, migraineurs had significant fALFF increases in bilateral ventral posteromedial (VPM) thalamus and brainstem encompassing rostral ventromedial medulla (RVM) and trigeminocervical complex (TCC). Regional fALFF values of bilateral VPM thalamus and brainstem positively correlated with disease duration, but not with migraine attack frequency or Migraine Disability Assessment Scale score.

**Conclusions:**

We have provided evidence for abnormal LFOs in the brainstem including RVM/TCC and thalamic VPM nucleus in migraine without aura, implicating trigeminothalamic network oscillations in migraine pathophysiology. Our results suggest that enhanced LFO activity may underpin the interictal trigeminothalamic dysrhythmia that could contribute to the impairments of pain transmission and modulation in migraine. Given our finding of increasing fALFF in relation to increasing disease duration, the observed trigeminothalamic dysrhythmia may indicate either an inherent pathology leading to migraine headaches or a consequence of repeated attacks on the brain.

## Background

Migraine is a disabling and common neurological disorder with a very high prevalence in the general population, manifested by recurrent pulsating headache of moderate-to-severe intensity, nausea/vomiting, and hypersensitivities to light, odor, sound, and touch [1]. Its fundamental pathophysiological mechanism has not been fully explicated; however, mounting evidence strongly suggests that migraine is a cortical brain disorder resulting from a dysfunctional neurolimbic pain networks [2, 3].

Recent advances in neuroimaging techniques have enormously contributed to our understanding of migraine pathophysiology. Specifically, morphometric MRI studies showed volume or cortical thickness changes of the brain, indicating macrostructural abnormalities of the central pain matrix in migraineurs [4–7]. Diffusion tensor imaging investigations exhibited alterations of the microstructural integrity and structural connectivity in pain processing regions including trigemino-thalamo-cortical pathway in migraine [5, 6, 8, 9]. Notably, a number of task-specific functional MRI (fMRI) studies have consistently revealed aberrant brain responses to visual, olfactory, and painful cutaneous stimuli, and lack of normal habituating response between migraine attacks [5, 6, 10]. Identification of the underlying mechanisms that give rise to sensory hypersensitivities and that provoke migraine headaches in response to sensory stimuli could help to improve our understanding of neural dysfunction in migraine pathophysiology.

Currently, resting-state fMRI has been increasingly used to evaluate large-scale functional connectomes at the whole-brain network level on the basis of temporally correlated fluctuations of blood oxygen level-dependent (BOLD) signals in the low-frequency range of 0.01–0.1 Hz. Because resting-state fMRI reliably establishes distinct corticocortical and corticosubcortical networks, it is extensively utilized to noninvasively assess abnormal functional networks in brain disorders including migraine [10, 11]. Previous resting-state fMRI studies successfully identified functional dysconnectivity of the cortical and subcortical regions within the nociceptive and somatosensory pathways during the different phases of migraine headache [6, 10–13]. Furthermore, these studies unveiled disruptions of various functional networks such as default mode network, dorsal attention network, central executive network, and salience network in migraineurs [5, 6, 10, 12, 13]. Collectively, findings from fMRI studies point to a hyperexcitable state as well as impairments in functional networks and multisensory integration, leading to hypersensitivity to sensory stimuli and pain processing abnormalities in migraine [5].

While most resting-state fMRI studies concentrated on the spatial properties of temporally correlated low-frequency oscillations (LFOs), commonly referred to as functional connectivity, it is also worthwhile to explore local brain activity by quantifying the amplitude of LFOs. Recently, a new analytic approach called ‘amplitude of low-frequency fluctuation (ALFF)’ has been developed and validated to detect functional connectivity changes in brain disorders [14]. ALFF estimates the total power of BOLD signals in the low-frequency range, whereas fractional ALFF (fALFF) stands for the relative contribution of LFOs by calculating the power within the low-frequency range divided by the total power in the whole detectable frequency range [15]. Relative to ALFF, fALFF is less sensitive to physiological noise, and considered to enhance the specificity and sensitivity in uncovering local brain activity [15]. Notwithstanding the increased recognition of LFOs in resting-state fMRI, the amplitude and spatial properties of spontaneous LFOs were assessed in only a few studies using ALFF or fALFF in migraineurs, with incongruent results between the studies [16–20].

In this study, we examined the spatial distribution of fALFF in a homogenous group of migraine patients without aura compared with control subjects. We predicted that LFO activity would be disturbed in migraineurs, resulting in an abnormal spontaneous brain activity within the central pain matrix during the interictal state. This abnormality in turn could be reflected in the disease severity of the patients, as defined by their clinical variables (e.g., migraine attack frequency, disease duration).

## Methods

### Participants

Forty-nine migraine patients were prospectively enrolled from the outpatient headache clinic of Korea University Guro Hospital. Patients had to meet the following inclusion criteria: (a) episodic migraine without aura according to the International Classification for Headache Disorders (ICHD-III beta); (b) 18–55 years of age; (c) no abnormalities on physical and neurological examinations; (d) no white matter hyperintensities on T2-weighted and fluid attenuated inversion recovery (FLAIR) images; and (e) free of migraine headache for at least 72 h before and 24 h after the scanning. Only three male patients were recruited during the study period. Given a high disproportion of female to male patients and the possible sex-specific differences in brain structures as well as functional connectivity in migraineurs [21], only female patients were selected for analysis to avert sex-linked bias. Patients were excluded if they had a history of psychiatric disease, neurological disease other than migraine, chronic systemic disease, or alcohol abuse. Since functional networks can be potentially influenced by topiramate or valproate [22, 23], the frequently used preventive medications for migraine, patients who had a history of receiving any preventive therapy for migraine were excluded as well. Demographics and the following clinical variables were acquired through interviewing of the patients and review of medical charts: age of migraine onset; disease duration; headache intensity measured by the visual analogue scale (VAS) score; laterality of headache (right, left, bilateral/alternating); attack frequency (headache days registered in a 3-month migraine diary prior to the MRI scanning); and the Migraine Disability Assessment Scale (MIDAS).

Thirty-three control subjects free from neurological and psychiatric diseases voluntarily participated in the current study. All controls reported no or extremely few spontaneous headaches, no family history of migraine, and no history of chronic pain disorders and alcohol abuse. The local ethics committee approved this study and all subjects gave written informed consent to participate in the study.

### Magnetic resonance imaging acquisition

Participants were scanned on a 3 Tesla MRI scanner with a standard 12-channel head coil (Siemens Trio, Erlangen, Germany). For identification of structural abnormalities, axial FLAIR and T2-weighted images (4 mm thickness), and oblique coronal FLAIR and T2-weighted images (3 mm thickness) were acquired. For reference purpose, a high-resolution T1-weighted image was obtained using a 3D magnetization-prepared rapid gradient-echo sequence (176 sagittal slices, TR/TE = 1780/2.34 ms, FOV = 256 mm^2^, matrix = 256 × 256, isotropic voxel dimensions = 1 mm^3^). For fALFF analysis, 245 volumes of 2D echo planar imaging were acquired (38 axial slices in interleaved-ascending order, TR/TE = 2000/30 ms, voxel size = 3.4 × 3.4 × 3.75 mm^3^). Participants were advised to rest motionlessly, think about nothing in particular, keep their eyes closed, and stay awake during the scanning. Participants with poor image quality from excessive head motion or image distortion due to various artifacts were excluded from the analysis.

### Image processing and fALFF computation

MRI data processing and fALFF computation were carried out using CONN-fMRI (version 19b, https://www.nitrc.org/projects/conn/), a comprehensive toolbox for resting-state fMRI analysis based on SPM12 (http://www.fil.ion.ucl.ac.uk/spm). Default preprocessing pipeline included: (a) eliminating the first five volumes for steady-state magnetization equilibrium; (b) slice-timing correction; (c) head motion estimation and correction; (d) direct segmentation of structural and functional images and spatial normalization into the Montreal Neurological Institute (MNI) space; (e) resampling to 2 mm^3^ isotropic voxels; and (f) smoothing with a 6 mm full-width half-maximum Gaussian kernel. Subject-specific nuisance regressors including 6 parameters acquired from rigid body head motion correction and their first derivatives were removed using a component-based noise correction strategy which uses principal component analysis approach in order to effectively eliminate physiological noises in resting-state fMRI data [24]. The white matter and cerebrospinal fluid signals were regressed out from the preprocessed functional images. Global signal regression was not done to prevent potential superfluous anti-correlations. Images with a framewise displacement value > 0.5 mm or global mean intensity changes > 3 standard deviations of mean were defined as outliers (https://www.nitrc.org/projects/artifact_detect/) and regressed out as well.

The preprocessed images were then temporally band-pass filtered (0.01–0.08 Hz) to diminish low-frequency drifts and physiological high-frequency cardiorespiratory noise. This procedure was simultaneously performed with nuisance regression in order to not reintroduce nuisance-related variations into a band-pass filtered time series. Computations of ALFF and fALFF were in accordance with previously illustrated methods by Zang et al. [14] and Zou et al. [15], respectively. In brief, the time series for a given voxel was first converted to the frequency domain using a Fast Fourier Transform. The square root of the power spectrum was computed and then averaged across a predefined frequency interval. This averaged square root was defined as ALFF at the given voxel, which is considered to reflect the absolute intensity of spontaneous brain activity [14]. fALFF is defined as the fraction of ALFF in a given frequency band (0.01–0.08 Hz) to the ALFF over the entire frequency range (0.01–0.25 Hz) detectable in the given signal [15]. The fALFF maps were finally *z*-transformed for standardization.

### Statistical analysis

The normalized fALFF maps were fed into the second-level analysis to compare the amplitude of LFOs between migraineurs and controls. Group-level statistical comparisons were assessed under the general linear model framework using random-effects two-sample *t*-test with age as a nuisance covariate to eliminate its contribution. Statistical significance was set at a height threshold of voxel-level *p* < 0.001 in conjunction with an extent threshold of cluster-level *p* < 0.05, corrected for multiple comparisons using familywise error (FWE). Relationships were explored between regional fALFF changes and clinical variables indicative of disease status in migraineurs. To this purpose, linear regression was conducted between the fALFF values extracted from the significant clusters (dependent variable) and disease duration and age (independent variables). Regional fALFF values were also correlated with headache frequency and MIDAS score by using Pearson correlation. Bonferroni correction was further applied to correct for multiple comparisons (*p* < 0.017 [0.05/3]). Statistical analyses were conducted using the Statistical Package for Social Sciences (version 25.0; IBM, Armonk, NY).

## Results

### Clinical characteristics

Of the 82 participants initially enrolled, five patients and two controls were excluded from the analysis due to either structural abnormalities or poor image quality on visual inspection of the MR images. Demographics and clinical characteristic of 44 patients with episodic migraine without aura (all females, mean age = 36.2 ± 8.8 years) and 31 controls (all females, mean age = 35.2 ± 9.2 years) are presented in Table 1. Results were expressed as mean ± standard deviation unless otherwise stated. Migraineurs and controls did not differ in age, education years, and Mini-Mental State Examination score. Mean age at migraine onset was 22.6 ± 6.4 years, and mean disease duration was 13.6 ± 5.8 years. Maximal pain intensity measured by VAS score was scale was 7.5 ± 1.7, and mean MIDAS score was 16.5 ± 7.8. Laterality of headache was reported as right-sided predominance in 16 patients, left-sided in 14, and bilateral/alternating sides in 14. Attack frequency based on the headache days registered in a 3-month migraine diary ranged from 3 to 24 (mean = 10.2 ± 5.2). Rescue medications that the patients habitually used for their migraine headaches consisted of triptans in 21 patients, ergotamine/caffeine tablets in 7, non-steroidal anti-infammatory drugs in 5, opioids in 2, and combination of above-mentioned drugs in 9.

**Table 1:** Demographics and clinical characteristics for migraineurs and controls

	Migraineurs (***n*** = 44)	Controls (***n*** = 31)	***p***-value (two-tailed)
Gender (female:male)	44:0	31:0	
Age (years)	36.2 ± 8.8 (range 18–54)	35.2 ± 9.2 (range 18–55)	0.640
Education years	14.3 ± 2.1	14.5 ± 1.6	0.632
MMSE score	28.9 ± 0.9	29.1 ± 1.1	0.307
Age of migraine onset (years)	22.6 ± 6.4 (range 13–34)		
Duration of migraine (years)	13.6 ± 5.8 (range 3–28)		
3-month headache frequency	10.2 ± 5.2 (range 3–24)		
Headache intensity (VAS score)	7.5 ± 1.7 (range 4–10)		
MIDAS score	16.5 ± 7.8 (range 4–33)		

Numerical data are presented as mean ± standard deviation. Group comparisons were made using two-sample *t*-test

*Abbreviations: MIDAS* Migraine Disability Assessment Scale, *MMSE* Mini-Mental State Examination, *VAS* visual analogue scale

### fALFF alterations

**Fig. 1:**
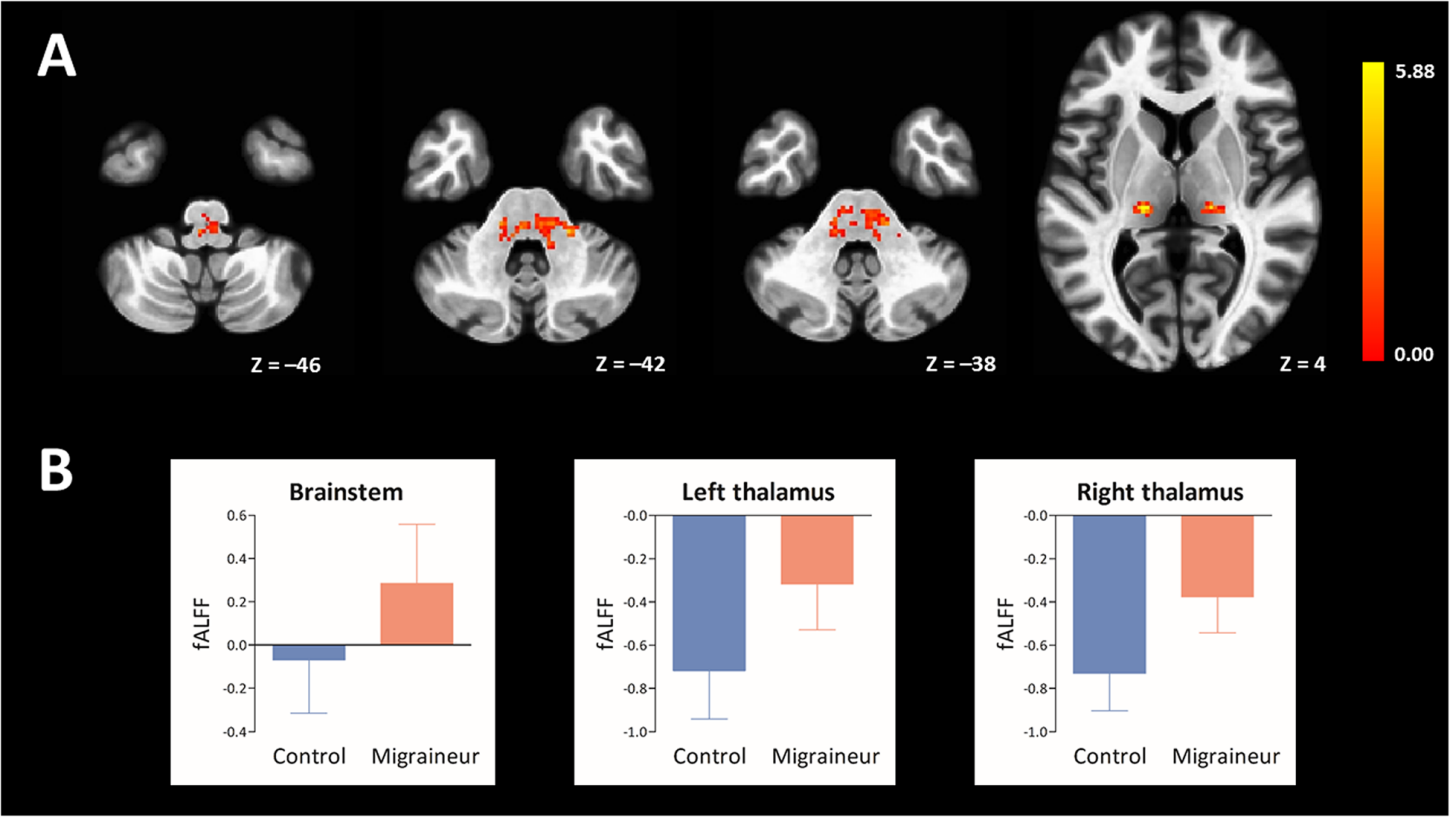
Brain regions of significant fALFF increases in migraine patients without aura compared to matched controls are rendered on the axial template images of the standard MNI brain (**A**). The left side of each MR image is the left side of the brain. The color bar indicates *t*-statistics. Mean (bars) and standard deviation (whiskers) for the fALFF of the brainstem and bilateral thalamic clusters in 31 controls and 44 migraine patients (two-sample *t*-test, all *p* < 0.001) (**B**)

**Fig. 2:**
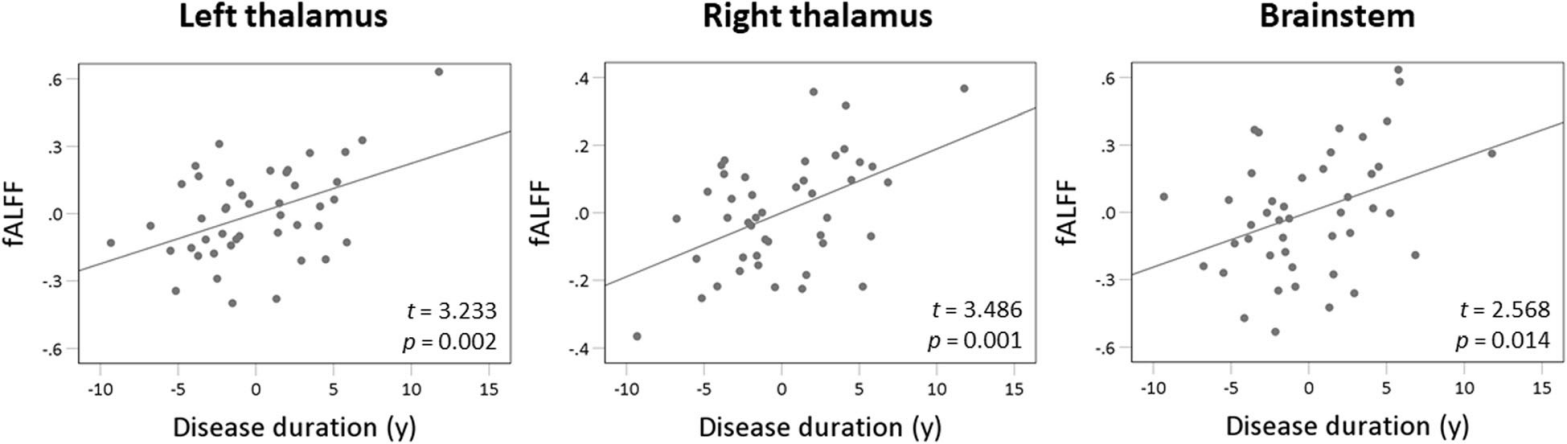
Scatter plot graphs of linear regression analysis showing that longer disease duration is significantly associated with an increased fALFF of bilateral thalamic and brainstem clusters (*p* < 0.017)

Compared to controls, migraineurs exhibited a significant increase of fALFF in the left ventral posteromedial (VPM) thalamus (MNI coordinate of local maxima = − 18/− 20/4, FWE-corrected *p* = 0.002, cluster volume = 1000 mm^3^, *t* = 5.04, *z* = 4.66), right VPM thalamus (MNI coordinate of local maxima = 22/− 20/4, FWE-corrected *p* < 0.001, cluster volume = 752 mm^3^, *t* = 5.78, *z* = 5.23), and brainstem (MNI coordinate of local maxima = 8/− 26/− 40, FWE-corrected *p* < 0.001, cluster volume = 3664 mm^3^, *t* = 5.88, *z* = 5.30) (Fig. 1). There was no region of decreased fALFF in migraineurs compared to controls. Longer disease duration in years of migraineurs was associated with an increased fALFF of left thalamic cluster (*β* = 0.613, *t* = 3.233, *p* = 0.002), right thalamic cluster (*β* = 0.662, *t* = 3.486, *p* = 0.001), and brainstem cluster (*β* = 0.516, *t* = 2.568, *p* = 0.014) (Fig. 2). Attack frequency registered in a 3-month migraine diary or MIDAS score did not significantly correlate with fALFF of the brainstem and bilateral thalamic clusters.

## Discussion

We attempted to determine alterations in intrinsic brain activity and to explore their relationships with clinical parameters reflecting disease severity in a homogenous cohort of migraine patients without aura using fALFF method that took into consideration the spatial distribution and amplitude of spontaneous LFOs. The main results are fALFF increases in bilateral VPM thalamus and brainstem regions incorporating rostral ventromedial medulla (RVM) and trigeminocervical complex (TCC) in migraineurs compared with controls. Longer disease duration was associated with an increased fALFF of the brainstem and bilateral VPM thalamus in migraineurs. Our finding of aberrant LFOs in the trigeminothalamic pathway of patients in the interictal state of migraine provides additional evidence to our understanding of the pathophysiological changes underlying migraine without aura.

### ALFF/fALFF alterations in migraine

ALFF/fALFF proved to be a reliable and reproducible data-driven approach that measures the amplitude of LFOs reflecting spontaneous neuronal activity at rest. The strengths of ALFF/fALFF method lie in the feasibility and simplicity of the analysis, remarkably high temporal stability [25], and excellent long-term inter-session test-retest reliability [26], thereby providing a robust marker of inter-individual and group differences in spontaneous LFOs [15, 27]. With respect to subsets of frequency bands, fALFF of slow-5 (0.01–0.027 Hz) and slow-4 (0.027–0.073 Hz) bands primarily reflect neuronal activity of the grey matter, while slow-4 band fALFF was higher throughout the thalamus, basal ganglia, and sensorimotor regions compared to slow-5 band fALFF. Slow-3 (0.073–0.198 Hz) and slow-2 (0.198–0.250 Hz) fALFF are mainly associated with white matter signal and physiological noise (respiratory and aliased cardiac signals) [27]. There is an ongoing debate as to whether resting-state fMRI could actually capture neuronal activity of the white matter, and thus, fMRI activation within the white matter remains contentious and warrants further investigation [28]. Furthermore, because ALFF is liable to be more vulnerable to pulsatile artifacts in the vicinity of blood vessels and ventricles, fALFF analysis is more effective than ALFF in detecting spontaneous LFOs in the periventricular and subcortical regions [27]. It seems therefore reasonable to adopt fALFF analysis using the low-frequency range of 0.01–0.08 Hz in our migraine patients in which LFO alteration would be expected in the subcortical and cortical grey matter such as thalamus and somatosensory cortex.

Alteration of fALFF reflects dysfunction of the local brain activity: increased fALFF found in migraineurs may represent an excitatory or facilitating process such as enhanced thalamocortical oscillations [17], whereas reduced fALFF can be interpreted as a functional inhibition of the brain regions and networks including default mode network, sensorimotor network, and dorsal attention network. There are, to our knowledge, 5 publicly available studies that explored ALFF/fALFF changes in migraineurs; however, their main results are conflicting [16–20]. The first study found ALFF decreases in the prefrontal and rostral anterior cingulate cortices, and ALFF increases in the right thalamus, suggesting interictal dysfunctional interaction between nociceptive processing and descending pain modulatory pathways in migraine without aura [16]. Another study investigating the same paradigm identified a significant reduction in ALFF in the primary somatosensory and premotor cortices in migraine without aura [20]. Significant ALFF/fALFF changes were also found in multiple brain regions linked to pain- and cognition-related functional networks in migraineurs compared to controls [18]. In a longitudinally designed study, migraine patients without aura had ALFF increases in the posterior insula and putamen/caudate, and ALFF reductions in the brainstem region of RVM/TCC [19]. Interestingly, reduced ALFF in the RVM/TCC in migraineurs was normalized following a 4-week’s acupuncture treatment, implying that impairment of the ascending nociceptive pathway and descending pain modulatory system in the brainstem might be implicated in neural pathophysiology of migraine [19]. In a seminal work by Hodkinson et al. [17], migraineurs exhibited an increase in fALFF of slow-5/4 bands (0.010–0.073 Hz) principally in the midline grey matter structures including thalamus, ventral diencephalon/hypothalamus, supplementary motor area, and anterior cingulate cortex, and a decrease in fALFF in bilateral prefrontal cortices. The study demonstrated the presence of aberrant LFOs in the thalamocortical network of migraine patients between headache attacks, implicating an abnormal interictal state of thalamocortical dysrhythmia in the pathogenetic mechanism of migraine [17]. Our findings of aberrant LFOs in the thalamus and brainstem nuclei in migraineurs are in line with those of aforementioned studies [16, 17, 19], although there is some divergence between the studies in whether the change is related with a decrease or an increase in ALFF/fALFF and in which thalamic nuclei are affected. However, we failed to replicate previous findings of fALFF changes in cortical regions involved in pain networks as well as affective and cognitive domains of pain [16–20]. The inconsistencies cannot be appropriately explained but might be partly attributed to genetic heterogeneity, different sample size, and methodological heterogeneity, particularly in image processing procedure, analysis software, and statistical inference. Confounding factors and clinical complexities within the patient population such as different migraine subgroups and sex difference, could also account for the inconsistent results between the studies.

### Brainstem nuclei

Recent advances in experimental and human functional imaging studies conceptualize migraine as a disorder of nociceptive circuits and multisensory network gain and plasticity [2, 29]. Specifically, dysfunction of the diencephalic and brainstem nuclei that modulate the activation of the trigeminovascular system, and their connections to other key centers of central pain matrix may lead to the predisposition and generation of headache and other concomitant symptoms of migraine [2, 3, 29]. In our study, migraineurs had a significant increase in fALFF in the brainstem encompassing RVM and TCC, well-known structures involved in nociceptive processing and migraine pathophysiology. RVM is the primary output structure mediating descending pain modulatory system [30]. ON and OFF cells within the RVM are activated on the onset and offset of noxious stimulation, and are considered to facilitate and suppress nociceptive transmission, respectively [31]. Increased ON cell and reduced OFF cell activities were observed in pain models including migraine [32, 33]. The first central relay for craniofacial pain is the TCC, which project directly or indirectly to the brain structures involved in the sensory/discriminative and affective/motivational aspects of pain, as well as to the structures involved in the descending pain modulation and autonomic outflow [2, 29, 34]. Physiological and tracing studies have proposed that activation of TCC and its connections (e.g., locus coeruleus, periaqueductal grey, hypothalamus, thalamus) could not only contribute to the perception of migraine headache, but also to affective, cognitive, and autonomic symptoms accompanied by migraine episode [2, 29]. Positron emission tomography studies unveiled neuronal activation in the brainstem, especially the dorsolateral pons, during spontaneous migraine attack [35, 36] and induced migraine [37]. Similarly, event-related fMRI studies disclosed BOLD signal increases in the brainstem encompassing the trigeminal nucleus or dorsal pons during migraine attack in response to nociceptive stimulation, highlighting the importance of TCC abnormality in the predisposition and generation of migraine headache [38–40]. Both macrostructural (e.g., volume reduction, shape deformation) and microstructural (e.g., increased mean diffusivity) abnormalities of the brainstem including trigeminal nucleus, dorsolateral pons, and periaqueductal grey were demonstrated in migraineurs in morphometric MRI and diffusion tensor imaging studies, respectively [41, 42]. Our finding of aberrant LFOs in the brainstem including RVM and TCC in migraineurs is in parallel with those of aforementioned studies, supporting the concept that the disturbed homeostasis of the trigeminovascular nociceptive pathway in the brainstem has a crucial role in the neurobiological mechanism of this disorder [2, 3, 29].

### Ventral posteromedial thalamic nucleus

The thalamus is the key center for conveying ascending nociceptive information from the peripheral nervous system to the cortex. Thalamic abnormalities in migraine has been well established in numerous animal studies and neurophysiological as well as human imaging studies such as volumetric MRI, diffusion tensor imaging, fMRI, and MR spectroscopy [43]. Particularly, trigeminovascular neurons send glutamatergic processes to the VPM thalamic nucleus, and VPM neurons are considered to principally relay sensory nociceptive information to the higher cortical pain processing regions of primary and secondary somatosensory cortices [2, 29, 44]. Involvement of VPM nucleus was supported by a few MRI studies showing structural and functional changes of the VPM nucleus in migraineurs. Migraineurs were found to have lower fractional anisotropy and shorter T1 relaxation time in the thalamic region corresponding to the VPM nucleus, indicating microstructural abnormality of VPM nucleus in migraine [8, 45]. Aberrant thalamocortical connectivity was identified between thalamic seed in close proximity to the VPM nucleus and pain modulating as well as pain encoding regions during spontaneous migraine attacks compared to interictal period [35].

The role of VPM nucleus in migraine pathophysiology has been emphasized in electrophysiological studies that evaluated the influence of migraine medications on thalamic VPM neurons in rats [46–49]. More specifically, acute medications such as triptans [46] and calcitonin gene-related peptide receptor antagonists [49], and preventive medications such as valproate [47] and topiramate [48] are able to inhibit nociceptive trigeminothalamic inputs in the VPM, strongly suggesting that VPM nucleus has a pivotal role in the modulation of pain transmission in migraine and could be a promising pharmacological target for migraine medications. Together, our finding of abnormal LFOs in the region of thalamic VPM nucleus in migraineurs accords with previous studies, further supporting the notions that VPM nucleus is primarily involved in migraine-relevant pain network, and its dysfunction may contribute to the generation and facilitation of migraine headache.

### Limitations and future perspectives

Our study has several inherent limitations that should be addressed. First, the sample sizes for both groups were small, which potentially limits the statistical robustness and reproducibility and reliability of the results [50, 51]. Second, since our patients were recruited from a tertiary referral hospital, they may not represent the general migraine population. In addition, only female migraine patients without aura were selected for statistical analysis. Given the likely differences in functional connectivity between female and male patients, and between migraine patients with aura and those without [21], our results could not be generalized to the entire patient population and should be confined to female migraine patients without aura. Third, the cross-sectional design of our study limits interpretation of the results regarding the directionality and causal relationship. Based on the compelling evidence of trigeminothalamic involvement in migraine, it is plausible that abnormal trigeminothalamic LFOs indicates an inherent pathology causing migraine headache. Conversely, given our finding of increasing fALFF in proportion to disease duration, we speculate that abnormal trigeminothalamic LFOs might be the consequence of repeated headaches or long-standing disease burden, and that LFO alteration could be used as a surrogate marker for the disease progression in migraine. Further prospective longitudinal studies would provide a clue to the disentanglement of causal relationships between spontaneous brain activity and disease progression. Fourth, our fMRI data utilized the relatively low sampling rate (TR = 2 s) without simultaneous measurement of cardiorespiratory activity that might generate artifactual signals in the low-frequency band; therefore, the potential influence of this physiological process on BOLD signal cannot be entirely eliminated [52]. However, a previous work demonstrated that regional differences in fALFF were not remarkably influenced by breath-holding process that strenuously manipulates the vascular contributions to the BOLD signal [27]. Moreover, this drawback is possibly mitigated by the fact that we analyzed fALFF instead of ALFF, which has proved to be more efficient than ALFF in capturing spontaneous LFOs of the subcortical and periventricular regions [27]. Last, our patients have taken a variety of prescribed antimigraine drugs for many years, which may compromise the results. The specific effects of different antimigraine drugs on intrinsic brain activity should be comprehended and applied to the future resting-state fMRI research in migraine.

## Conclusion

Converging evidence suggest that the sensory nervous system plays a critical role in the generation and predisposition of migraine headache. Herein, we demonstrated abnormal LFOs in the brainstem including RVM/TCC and thalamic VPM nucleus, implicating trigeminothalamic network oscillations in the pathophysiology underlying migraine without aura. Our results suggest that enhanced LFO activity may underpin the presence of interictal trigeminothalamic dysrhythmia that could contribute to the impairments of pain transmission and modulation in migraine. Given the finding of increasing fALFF in relation to increasing disease duration, the observed trigeminothalamic dysrhythmia may indicate either an inherent pathology leading to migraine headaches or a consequence of repeated attacks on the brain.

## Data Availability

The datasets used and/or analyzed during the current study are not publicly available due to privacy concerns, but are available from the corresponding author on reasonable request.

## Abbreviations

ALFF: Amplitude of low-frequency fluctuation
BOLD: Blood oxygen level-dependent
fALFF: fractional amplitude of low-frequency fluctuation
fMRI: functional magnetic resonance imaging
LFO: Low-frequency oscillation
MIDAS: Migraine Disability Assessment Scale
MNI: Montreal Neurological Institute
RVM: Rostral ventromedial medulla
TCC: Trigeminocervical complex
VAS: Visual analogue scale
VPM: Ventral posteromedial
